# Electrodeposited Copolymers Based on 9,9′-(5-Bromo-1,3-phenylene)biscarbazole and Dithiophene Derivatives for High-Performance Electrochromic Devices

**DOI:** 10.3390/polym13071136

**Published:** 2021-04-02

**Authors:** Chung-Wen Kuo, Jui-Cheng Chang, Jeng-Kuei Chang, Sheng-Wei Huang, Pei-Ying Lee, Tzi-Yi Wu

**Affiliations:** 1 Department of Chemical and Materials Engineering, National Kaohsiung University of Science and Technology, Kaohsiung 80778, Taiwan; welly@nkust.edu.tw (C.-W.K.); bill4794@gmail.com (S.-W.H.); 2 Department of Chemical Engineering and Materials Engineering, National Yunlin University of Science and Technology, Yunlin 64002, Taiwan; d700215@gmail.com (J.-C.C.); leepeiying1018@gmail.com (P.-Y.L.); 3 Bachelor Program in Interdisciplinary Studies, National Yunlin University of Science and Technology, Yunlin 64002, Taiwan; 4 Department of Materials Science and Engineering, National Yang Ming Chiao Tung University, No. 1001 University Road, Hsinchu 30010, Taiwan; jkchang@nctu.edu.tw

**Keywords:** electrodeposition, absorption spectra, transmittance, electrochromic device, redox stability

## Abstract

A 1,3-bis(carbazol-9-yl)benzene derivative (BPBC) was synthesized and its related homopolymer (PBPBC) and copolymers (P(BPBC-*co*-BT), P(BPBC-*co*-CDT), and P(BPBC-*co*-CDTK)) were prepared using electrochemical polymerization. Investigations of polymeric spectra showed that PBPBC film was grey, iron-grey, yellowish-grey, and greyish-green from the neutral to the oxidized state. P(BPBC-*co*-BT), P(BPBC-*co*-CDT), and P(BPBC-*co*-CDTK) films showed multicolor transitions from the reduced to the oxidized state. The transmittance change (Δ*T*) of PBPBC, P(BPBC-*co*-BT), P(BPBC-*co*-CDT), and P(BPBC-*co*-CDTK) films were 29.6% at 1040 nm, 44.4% at 1030 nm, 22.3% at 1050 nm, and 41.4% at 1070 nm. The coloration efficiency (*η*) of PBPBC and P(BPBC-*co*-CDTK) films were evaluated to be 140.3 cm^2^ C^−1^ at 1040 nm and 283.7 cm^2^ C^−1^ at 1070 nm, respectively. A P(BPBC-*co*-BT)/PEDOT electrochromic device (ECD) showed a large Δ*T* (36.2% at 625 nm) and a fast response time (less than 0.5 s), whereas a P(BPBC-*co*-CDTK)/PEDOT ECD revealed a large *η* (534.4 cm^2^ C^–1^ at 610 nm) and sufficient optical circuit memory.

## 1. Introduction

Organic conjugated polymers have attracted more attention over the past two decades owing to their versatile applications in energy-stored devices [1,2], electrochromic devices [3,4,5,6,7], sensors [8,9,10], catalysts [11,12,13], solar cells [14,15], field effect transistors [16,17], and light-emitting diodes [18,19,20]. Today, many practical applications of electrochromic technologies, including self-tunable eyewear, auto-dimmer rearview mirrors, smart textiles, and electronic paper, are presented in our daily lives [21,22]. Electrochromic technologies are further used in flexible technical fields such as wearable electronics, camouflage, and curved windows. Electrochromic materials of electrochromic devices can be classified as inorganic and organic electrochromic materials [23,24]. Although inorganic electrochromic materials display substantial advantages concerning their high transmittance variation and high thermal stability, organic electrochromic materials exhibit many advantages over inorganic electrochromic materials owing to their fast response time, multi-color electrochromism, satisfactory switching reproducibility, and tunable bandgaps with changes of polymeric structures [25]. Organic, conjugated polymers belong to organic electrochromic materials. Polyimides [26], polycarbazoles [27,28,29], polythiophenes [30,31], polyindoles [32,33], poly(dioxythiophene)s [34,35], polytriphenylamines [36,37], and polythiadiazoles [38] are the main organic conjugated polymers used in several organic ECDs. Among them, polycarbazoles have attracted increased attention owing to their conspicuous absorption in the UV–Vis zone, good photo- and electroactive properties, and high hole-transporting ability. Niu et al. reported the spectroelectrochemical properties of six carbazole-containing polymers; the color of PDCB-DF film changed from light yellow to violet red from the neutral to the oxidized state [39]. PDCB-DTC-DF displayed a high Δ*T* (74.4% at 1230 nm), a high *η* (365 cm^2^ C^–1^ at 1230 nm), and good electrochromic stability. Ak et al. published the electrochromic properties of a multifunctional and solution-processable polymer (PCzRY) [40]. PCzRY film displayed a high transmittance change (70%), a high *η* (678 cm^2^/C), and a satisfactory cycling stability (92%). PCzRY film changed from transparent to aquamarine during its redox procedure. Poly(3,4-ethylenedioxythiophene) (PEDOT) and its derivatives such as PProDOT and PEDOT:PSS are poly(dioxythiophene) derivatives. Poly(dioxythiophene)s display lower bandgaps and higher the highest occupied molecular orbital (HOMO) energy levels than those of polythiophenes [41]. Moreover, colored PEDOT, PProDOT, and PEDOT:PSS can be oxidized into a bleached state. Therefore, PEDOT, PProDOT, and PEDOT:PSS can be categorized into the cathodic layers of ECDs [42]. On the other hand, the incorporation of a bithiophene unit into the polymeric backbones can modify the color category and conjugation degree. Two thiophene units in a 4H-cyclopenta[1,2-b:5,4-b’]dithiophene (CDT) ring are rigidified by a covalent carbon, which increases the planarity of the backbone. The incorporation of a ketone group in cyclopentadithiophene ketone (CDTK) presents lower electrochemical bandgaps than that of CDT.

In this study, a carbazole derivative (9,9′-(5-bromo-1,3-phenylene)biscarbazole (BPBC)) was synthesized, and a BPBC-containing homopolymer (PBPBC) and three BPBC-containing copolymers (P(BPBC-*co*-BT), P(BPBC-*co*-CDT), and P(BPBC-*co*-CDTK)) were synthesized electrochemically. Upon halogen (bromo group) substitution, BPBC displayed a higher *E*_onset_ as compared to unsubstituted 1,3-bis(carbazol-9-yl)benzene, indicating a decrease in the HOMO energy level. A PBPBC, P(BPBC-*co*-BT), P(BPBC-*co*-CDT), or P(BPBC-*co*-CDTK) film was employed as the anodic layer of the ECDs, and a PEDOT film was employed as the cathodic layer of the ECDs. The electrochromic phenomena and bleach–color kinetics of PBPBC, P(BPBC-*co*-BT), P(BPBC-*co*-CDT), or P(BPBC-*co*-CDTK) films and the spectral properties, redox cycling stabilities, and open circuit memory of PBPBC/PEDOT, P(BPBC-*co*-BT)/PEDOT, P(BPBC-*co*-CDT)/PEDOT, and P(BPBC-*co*-CDTK)/PEDOT ECDs were explored exhaustively.

## 2. Experimental

### 2.1. Materials

Carbazole, 1-bromo-3,5-difluorobenzene, *t*-BuOK, EDOT, BT, CDT, and CDTK were purchased from Tokyo Chemical Industry (Tokyo, Japan), Acros (Geel, Belgium), and Aldrich (St. Louis, MO, USA), and used as received. As shown in Table 1, P(BPBC-*co*-BT), P(BPBC-*co*-CDT), and P(BPBC-*co*-CDTK) films were electrodeposited using a feed molar ratio of BPBC/BT, BPBC/CDT, or BPBC/CDTK at 1/1. 

### 2.2. Synthesis of BPBC

**Figure 1:**
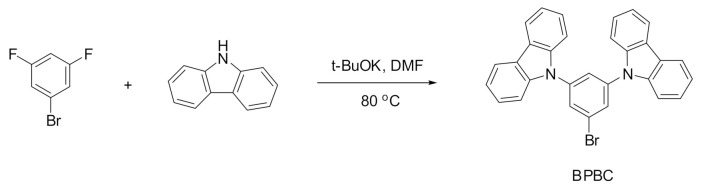
The synthetic procedure of 9,9′-(5-bromo-1,3-phenylene)biscarbazole (BPBC).

In an inert atmosphere, carbazole (0.836 g, 5 mmol) in 15 mL dry DMF (*N,N*-dimethylformamide) was added into a solution (*t*-BuOK (0.561 g, 5 mmol) dissolved in 15 mL dry DMF) dropwise and stirred for a half hour. Afterward, 1-bromo-3,5-difluorobenzene (0.463 g, 2.4 mmol) in 10 mL dry DMF was added into the mixture. The solution was further stirred at 140 °C for 1 day. After the reaction was completed (Figure 1), 300 mL of deionized water was poured into the mixture and the solid precipitate was filtered and dried under a vacuum. The crude product was purified using column chromatography (silica, eluent: DCM/hexane = 1/1). Yield: 67%. ^1^H NMR (500 MHz, CDCl_3_): *δ* = 7.36 (t, J = 7.6 Hz, 4H, carbazole-H), 7.49 (t, J = 7.6 Hz, 4H, carbazole-H), 7.58 (d, J = 7.6 Hz, 4H, carbazole-H), 7.82 (t, J = 1.9 Hz, 1H, benzene-H), 7.89 (d, J = 1.9 Hz, 2H, benzene-H), 8.17 (d, J = 7.6 Hz, 4H, carbazole-H). ^13^C NMR (125 MHz, CDCl_3_): *δ* = 109.6, 120.5, 120.7, 123.8, 123.9, 124.1, 126.3, 128.6, 140.3, 140.4. Elem. Anal. Calcd. for C_30_H_19_BrN_2_: C, 73.93%; H, 3.93%; N, 5.75%. Found: C, 73.81%; H, 3.87%; N, 5.63%.

### 2.3. Preparation of Electrolyte and Assembly of the ECDs

**Figure 2:**
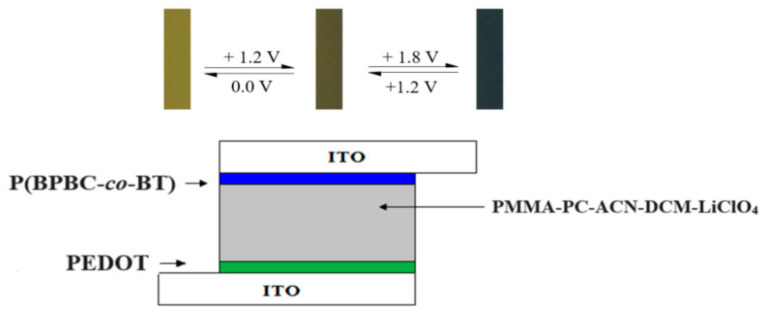
Schematic diagrams of the P(BPBC-*co*-BT)/PEDOT device.

The electrolyte of the ECDs was prepared using 0.4 g of PMMA, 0.3 g of LiClO_4_, 1.1 g of PC, and 2.5 mL of ACN based on previous procedures [43]. PBPBC/PEDOT, P(BPBC-*co*-BT)/PEDOT, P(BPBC-*co*-CDT)/PEDOT, and P(BPBC-*co*-CDTK)/PEDOT dual type ECDs were assembled using the electrolyte as the separation layer between anodic and cathodic polymer layers (Figure 2). The anodic polymer layers (PBPBC, P(BPBC-*co*-BT), P(BPBC-*co*-CDT), and P(BPBC-*co*-CDTK) films) were prepared potentiodynamically in a potential range from 0.0 to 1.6 V. The cathodic PEDOT layer was prepared potentiostatically at 1.4 V on indium tin oxide (ITO) glass. The active electrode area of the ECDs was 1.5 cm^2^.

### 2.4. Electrochemical, Optical, and Electrochromic Properties

Spectroelectrochemical properties of the polymers and ECDs were measured using an electrochemical analyzer (CHI627E, (CH Instruments, Austin, TX, USA)) and a Jasco V-630 absorption spectrometer (JASCO International Co., Ltd., Tokyo, Japan). The cyclic voltammetry (CV) experiments were also carried out using the CHI627E electrochemical workstation in a three-electrode cell with a working electrode (ITO glasses), a counter electrode (Pt wire), and a reference electrode (Ag/AgCl). The three electrodes were dipped in 0.2 M LiClO_4_/ACN/DCM, where the volume ratio of ACN/DCM = 4/1.

## 3. Results and Discussion

### 3.1. Electrochemical Characterization

**Figure 3:**
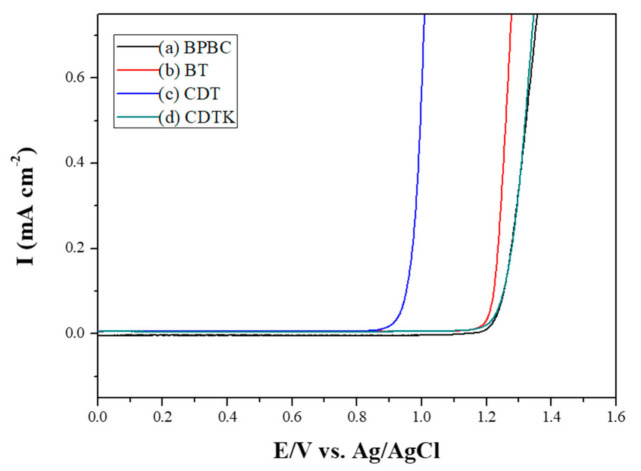
Electrooxidation curves of (**a**) 2 mM BPBC, (**b**) 2 mM BT, (**c**) 2 mM CDT, and (**d**) 2 mM CDTK in 0.2 M LiClO_4_ of ACN/DCM solution (ACN/DCM = 4:1, by volume) at 100 mV s^−1^.

As displayed in Figure 3, the *E*_onset_ (vs. Ag/AgCl) values of BPBC, BT, CDT, and CDTK were 1.26, 1.22, 0.95, and 1.25 V, respectively. The *E*_onset_ of BPBC was comparable to those of BT and CDTK, implying that the electron-withdrawing bromo substituted group increased the oxidized potential of 1,3-di(9H-carbazol-9-yl)benzene. The *E*_onset_ of CDT was smaller than that of CDTK, which can be attributed to the ketone group of CDTK increasing the *E*_onset_ significantly.

**Figure 4:**
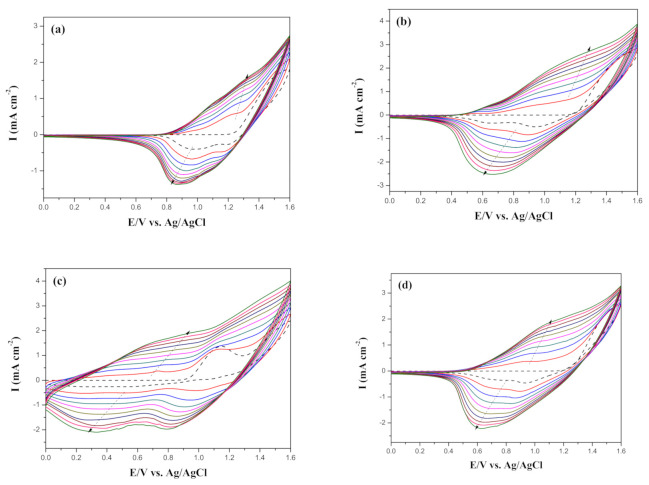
Electrochemical syntheses of (**a**) PBPBC, (**b**) P(BPBC-*co*-BT), (**c**) P(BPBC-*co*-CDT), and (**d**) P(BPBC-*co*-CDTK) in 0.2 M LiClO_4_ of ACN/DCM solution (ACN/DCM = 4:1, by volume) at 100 mV∙s^−1^.

Figure 4 shows the electro-synthesized curves of a neat BPBC monomer and the mixtures of two monomers (BPBC + BT, BPBC + CDT, and BPBC + CDTK) in 0.2 M LiClO_4_/ACN/DCM (ACN/DCM = 4:1, by volume). During the potential scan of CV in the anodic region, two semi-reversible oxidized waves were observed in Figure 4, which could be assigned to the formation of a cation radical and the quinoid-like dication of bicarbazole segments [44]. As the electrosynthesized curves increased with the increasing number of times, the PBPBC, P(BPBC-*co*-BT), P(BPBC-*co*-CDT), and P(BPBC-*co*-CDTK) films could be presented on the ITO substrates, manifesting that PBPBC, P(BPBC-*co*-BT), P(BPBC-*co*-CDT), and P(BPBC-*co*-CDTK) films were coated on the ITO glasses [45].

**Figure 5:**
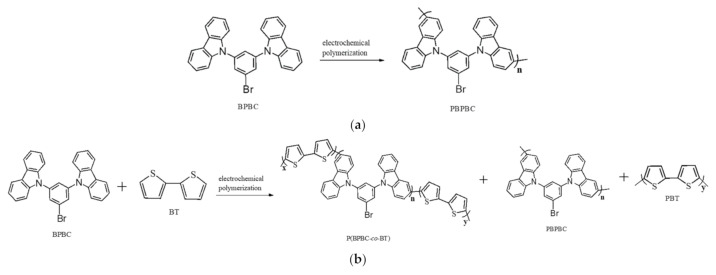

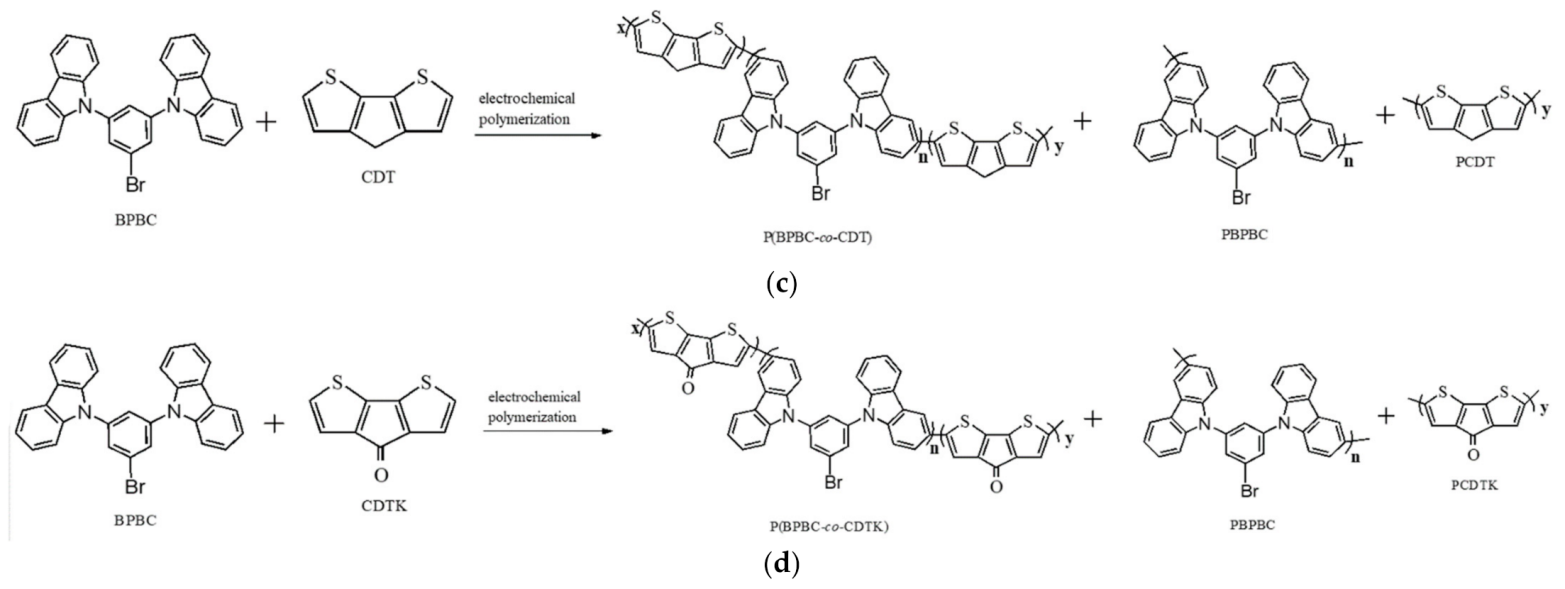
Electrochemical polymerizations of (**a**) PBPBC, (**b**) P(BPBC-*co*-BT), (**c**) P(BPBC-*co*-CDT), and (**d**) P(BPBC-*co*-CDTK).

As displayed in Figure 4b–d, the cyclic voltammetric redox peaks and wave-profiles of P(BPBC-*co*-BT), P(BPBC-*co*-CDT), and P(BPBC-*co*-CDTK) films were dissimilar to those of PBPBC films, implying copolymer films were coated on the ITO surfaces (Figure S1 Supplementary Materials). Figure 5 revealed the schemes of electrosynthesis of PBPBC, P(BPBC-*co*-BT), P(BPBC-*co*-CDT), and P(BPBC-*co*-CDTK).

**Figure 6:**
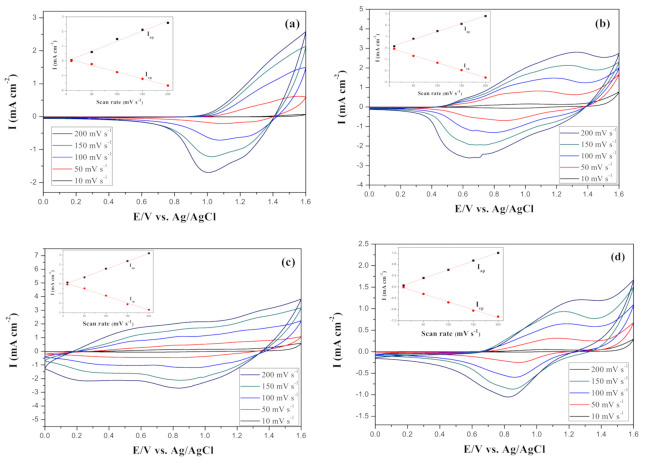
CV curves of (**a**) PBPBC, (**b**) P(BPBC-*co*-BT), (**c**) P(BPBC-*co*-CDT), and (**d**) P(BPBC-*co*-CDTK) films at different scan rates between 10 and 200 mV s^−1^ in 0.2 M LiClO_4_ of ACN/DCM solution (ACN/DCM = 4:1, by volume). Insets are the scan rate dependence of anodic and cathodic peak current densities of electrodes.

The electrochemical properties of PBPBC, P(BPBC-*co*-BT), P(BPBC-*co*-CDT), and P(BPBC-*co*-CDTK) films were characterized by sweeping in monomer-free electrolyte at various scan rates. Figure 6 exhibited well-defined reversible oxidization and reduction processes for PBPBC, P(BPBC-*co*-BT), P(BPBC-*co*-CDT), and P(BPBC-*co*-CDTK) films. The anodic and cathodic peak currents exhibited a linear growth with increasing scanning rates, disclosing that the electroactive species transferred during the oxidation-reduction reactions were nondiffusion-limited and PBPBC, P(BPBC-*co*-BT), P(BPBC-*co*-CDT), and P(BPBC-*co*-CDTK) were tightly attached to the ITO glasses [46].

### 3.2. Spectral Characterization of Electrodes

**Figure 7:**
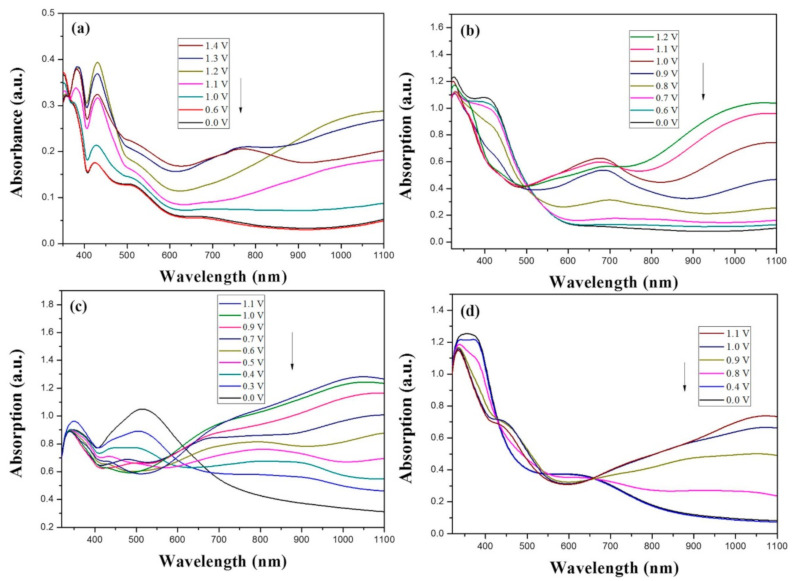
UV–Visible spectra of (**a**) PBPBC, (**b**) P(BPBC-*co*-BT), (**c**) P(BPBC-*co*-CDT), and (**d**) P(BPBC-*co*-CDTK) in 0.2 M LiClO_4_ of ACN/DCM solution (ACN/DCM = 4:1, by volume).

Figure 7 displayed the absorption spectra of PBPBC, P(BPBC-*co*-BT), P(BPBC-*co*-CDT), and P(BPBC-*co*-CDTK) electrodes at various voltages. PBPBC film displayed a large absorption band at 350 nm and a small peak at around 430 nm, which could be attributed to the π–π* transition and n–π* transition of PBPBC, respectively. When the voltage was slowly increased, the absorption band of the π–π* transition faded little by little, and the charged carrier bands ascended at ca. 380, 430, 750, and 1100 nm [47]. As the potential was increased to 1.3 V, the absorption band at 750 to 1100 nm began to drop slightly, implying the appearance of a bipolaron band caused by further oxidations of the 1,3-bis(carbazol-9-yl)benzene unit [48]. P(BPBC-*co*-BT) film displayed a π–π* transition shoulder of the BT ring at 420 nm, with the charged carrier bands of P(BPBC-*co*-BT) located at 680 and 1050 nm. The oxidation of polythiophene segments occurred at the beginning, and then, at a slightly higher potential, bicarbazole segments were oxidized in two steps. 

The π–π* transition peak of P(BPBC-*co*-CDT) film shifted bathochromically to 510 nm, indicating the cyclopenta[2,1-b:3,4-b’]dithiophene unit was more planar than the BT ring. Compared to P(BPBC-*co*-CDT) film, the π–π* transition of P(BPBC-*co*-CDTK) shifted hypsochromically to 370 nm, which may be ascribed to the low polymerization degree or low planarity of P(BPBC-*co*-CDTK). The charged carrier bands of P(BPBC-*co*-CDTK) film were located at more than 700 nm.

Table 2 shows the photo images, L*, a*, b*, *x*, and *y* values of PBPBC, P(BPBC-*co*-BT), P(BPBC-*co*-CDT), and P(BPBC-*co*-CDTK) at various voltages in solutions. PBPBC film was grey (0.0 V) in the neutral state, iron grey (0.4 V), yellowish-grey (0.8 V), and greyish-green (1.2 V) in the oxidized state. P(BPBC-*co*-BT), P(BPBC-*co*-CDT), and P(BPBC-*co*-CDTK) films also showed multicolor transitions from the neutral to the oxidized state. P(BPBC-*co*-BT) film was yellowish-brown, khaki, greyish-green, and bluish-green at 0.0, 0.4, 0.8, and 1.2 V, respectively. P(BPBC-*co*-CDT) film was purplish-black, dark orchid, greyish-blue, and Prussian blue at 0.0, 0.4, 0.8, and 1.1 V, respectively. P(BPBC-*co*-CDTK) was charcoal-grey, dim-grey, grey, and greyish-yellow at 0.0, 0.4, 0.8, and 1.1 V, respectively. The Commission Internationale de I’Eclairage (CIE) diagrams of PBPBC, P(BPBC-*co*-BT), P(BPBC-*co*-CDT), and P(BPBC-*co*-CDTK) films in neutral and oxidized states are shown in Table 2.

The bandgap (*E*_g_) of the PBPBC film determined using the absorption edge values of the UV spectrum was 2.64 eV [49]. The *E*_onset(ox)_ of PBPBC (vs. Ag/AgCl) was 1.25 V, and the *E*_FOC_ value obtained from Fc/Fc^+^ was 0.81 V. The *E*_onset(ox)_ (vs. *E*_FOC_) was evaluated as 0.44 V. The HOMO energy level of PBPBC was estimated using the *E*_onset_ and the energy level of the Fc/Fc^+^ redox couple (–4.8 eV below the vacuum level) [50,51]. The lowest unoccupied molecular orbital (LUMO) energy level of PBPBC (vs. vacuum level) was calculated by the addition of *E*_g_ from the HOMO level (–5.24 eV), and the *E*_LUMO_ of PBPBC was –2.60 eV. 

### 3.3. Kinetics Studies of Polymeric Coloring and Bleaching

**Figure 8:**
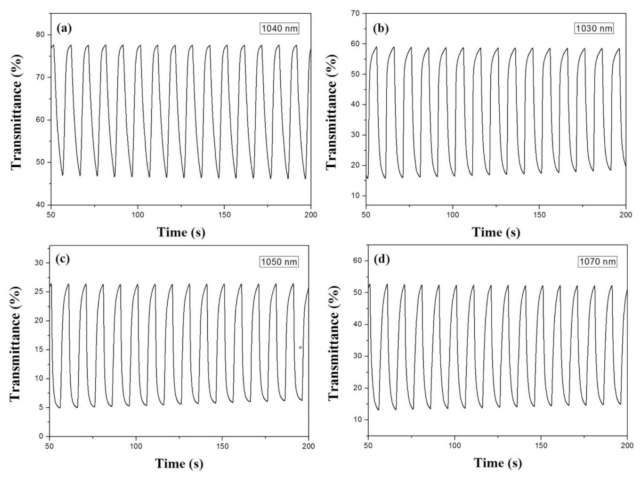
Optical contrast of (**a**) PBPBC, (**b**) P(BPBC-*co*-BT), (**c**) P(BPBC-*co*-CDT), and (**d**) P(BPBC-*co*-CDTK) in 0.2 M LiClO_4_ of ACN/DCM solution (ACN/DCM = 4:1, by volume) with a residence time of 5 s.

Figure 8 showed the dynamic coloring and bleaching profiles of PBPBC, P(BPBC-*co*-BT), P(BPBC-*co*-CDT), and P(BPBC-*co*-CDTK) films in solutions between 0.0 and 1.2 V (or 1.1 V) with a time interval of 5 s. The bleaching and coloring switching times (*τ*_b_ and *τ*_c_) were evaluated at 90% of total transmittance change, and the values are summarized in Table 3. The *τ*_b_ and *τ*_c_ of PBPBC, P(BPBC-*co*-BT), P(BPBC-*co*-CDT), and P(BPBC-*co*-CDTK) films at various wavelengths were calculated to be 0.4–2.5 s in solutions. PBPBC, P(BPBC-*co*-BT), and P(BPBC-*co*-CDTK) had a faster coloration time (0.4 s at 430 nm, 1.0 s at 680 nm, and 0.7 s at 460 nm) compared to their bleaching time (1.5 s at 430 nm, 2.5 s at 680 nm, and 2.3 s at 460 nm), which could be attributed to a bulkier anion, such as ClO_4_^−^, being more likely to get trapped onto the polymeric chains [52]. However, P(BPBC-*co*-CDT) had a slower coloration time (2.5 s at 780 nm) compared to its bleaching time (1.5 s at 780 nm), inferring that a high planar CDT ring in the polymer backbone gave rise to a slower coloring velocity.

The transmittance changes between the bleached and colored states of PBPBC, P(BPBC-*co*-BT), P(BPBC-*co*-CDT), and P(BPBC-*co*-CDTK) films were 29.6% at 1040 nm, 44.4% at 1030 nm, 22.3% at 1050 nm, and 41.4% at 1070 nm at the second cycle. The Δ*T* of P(BPBC-*co*-BT) and P(BPBC-*co*-CDTK) films in near-infrared spectral zone were larger than that of PBPBC, inferring BT- and CDTK-containing copolymers presented higher Δ*T* in the near-infrared region than that of PBPBC homopolymer. The Δ*T* of PBPBC, P(BPBC-*co*-BT), P(BPBC-*co*-CDT), and P(BPBC-*co*-CDTK) in the near infrared (NIR) region was larger than those in the visible region, which could be ascribed to the emergence of a significant polaron and bipolaron upon oxidizing. Table 4 lists the comparison of Δ*T* with the reported polymers in solutions. P(BPBC-*co*-BT) displayed a higher Δ*T* than those reported for P(DiCP-*co*-CPTK2) at 890 nm [53], PITD-2 at 675 nm [54], and P2 at 779 nm [55]. However, the Δ*T* of P(BPBC-*co*-BT) was lower than those of DPPA-2SNS at 900 nm [56] and PI-6A at 573 nm [57].

The optical density (ΔOD) of PBPBC, P(BPBC-*co*-BT), P(BPBC-*co*-CDT), and P(BPBC-*co*-CDTK) at visible and NIR regions can be estimated using the following equation [58]:

(1) $$\Delta \mathrm{OD}=\log\left(\frac{T_{\mathrm{ox}}}{T_{\mathrm{re}d}}\right)$$

where *T*_red_ and *T*_ox_ indicate the transmittance of electrodes in the reduced and the oxidized state, respectively. Similar to the trend of Δ*T*, the PBPBC, P(BPBC-*co*-BT), P(BPBC-*co*-CDT), and P(BPBC-*co*-CDTK) showed higher ΔOD in near-infrared spectral regions than those in visible regions.

Another crucial criterion of electrochromism is the coloration efficiency (*η*), which can be calculated from the following equation [61]:

(2) $$\eta =\frac{\Delta \mathrm{OD}}{Q_{d}}$$

where *Q*_d_ stands for the injected/ejected charge as a function of electrode active area. As presented in Table 3, the *η* of PBPBC, P(BPBC-*co*-BT), P(BPBC-*co*-CDT), and P(BPBC-*co*-CDTK) films were evaluated to be 140.3 cm^2^ C^−1^ at 1040 nm, 130.0 cm^2^ C^−1^ at 1030 nm, 121.7 cm^2^ C^−1^ at 1050 nm, and 283.7 cm^2^ C^−1^ at 1070 nm, respectively. As shown in Table 4, P(BPBC-*co*-BT) displayed a larger *η* value than those reported for P(DiCP-*co*-CPTK2) at 890 nm [53], PITD-2 at 675 nm [54], P2 at 779 nm [55], and PI-6A at 573 nm [57], whereas the *η* of P(BPBC-*co*-BT) was comparable to that reported for DPPA-2SNS at 900 nm [56].

### 3.4. Photoluminescence of Polymers

**Figure 9:**
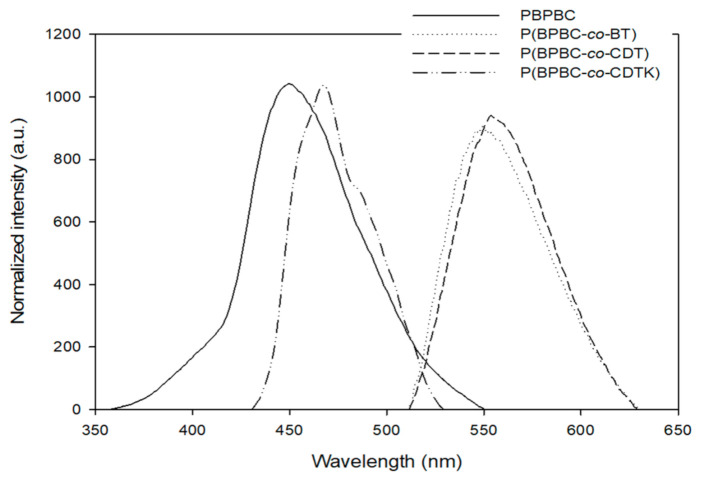
The photoluminescence (PL) spectra of PBPBC (*λ*_ex_ = 310 nm), P(BPBC-*co*-BT) (*λ*_ex_ = 400 nm), P(BPBC-*co*-CDT) (*λ*_ex_ = 400 nm), and P(BPBC-*co*-CDTK) (*λ*_ex_ = 310 nm) films.

Figure 9 shows the photoluminescence (PL) spectra of as-prepared polymeric films. The emission peaks of P(BPBC-*co*-BT) and P(BPBC-*co*-CDT) exhibited larger PL maxima than that of PBPBC, implying the incorporation of dithiophene derivatives (BT and CDT) in the polymer backbone gave rise to a bathochromic shift in PL spectra. However, P(BPBC-*co*-CDTK) displayed a hypsochromic shift in the PL maximum with respect to those of P(BPBC-*co*-BT) and P(BPBC-*co*-CDT), which may be ascribed to the electron-withdrawing ketone groups of P(BPBC-*co*-CDTK) changing the electronic distribution and decreasing π–π stacking or the planarity of the polymer backbone.

### 3.5. Spectral Characterization of ECDs

**Figure 10:**
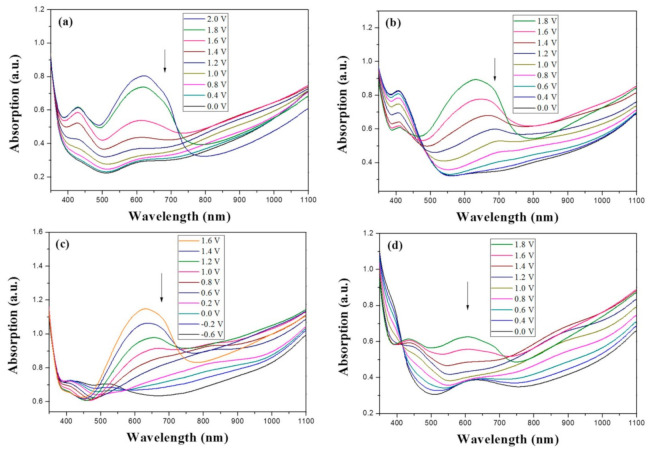
UV–Visible spectra of (**a**) PBPBC/PEDOT, (**b**) P(BPBC-*co*-BT)/PEDOT, (**c**) P(BPBC-*co*-CDT)/PEDOT, and (**d**) P(BPBC-*co*-CDTK)/PEDOT ECDs.

The ionic conductivity of the gel polymer electrolyte used in the sandwich-type ECDs was about 1.4 × 10^−3^ S/cm, and the PMMA-based composite electrolyte was transparent at a wide potential range. Figure 10 displays the absorption spectra of PBPBC/PEDOT, P(BPBC-*co*-BT)/PEDOT, P(BPBC-*co*-CDT)/PEDOT, and P(BPBC-*co*-CDTK)/PEDOT ECDs at numerous potentials.

PBPBC/PEDOT ECD did not display obvious peaks at wavelength less than 500 nm. P(BPBC-*co*-BT)/PEDOT, P(BPBC-*co*-CDT)/PEDOT, and P(BPBC-*co*-CDTK)/PEDOT ECDs showed peaks or shoulders at ca. 420, 510, and 380 nm at ca. 0.0 V, respectively, which were consistent with the π–π* (or n–π*) transition of P(BPBC-*co*-BT), P(BPBC-*co*-CDT), and P(BPBC-*co*-CDTK) in reduced states. In such situations, the PEDOT cathode was sorted as in its oxidation state and did not show distinct absorption bands in the UV–Vis region [62]. When the applied voltage was increased gradually, the anodic layers began to oxidize, and the cathodic layers began to reduce. Therefore, absorption bands of ECDs began to turn up at 610–630 nm, and the noticeable color of the four ECDs was blue at high potentials as shown in Table 5. Table 5 also shows the CIE diagrams of the four ECDs at bleached and colored states.

### 3.6. Kinetics Studies of ECDs’ Coloring and Bleaching

**Figure 11:**
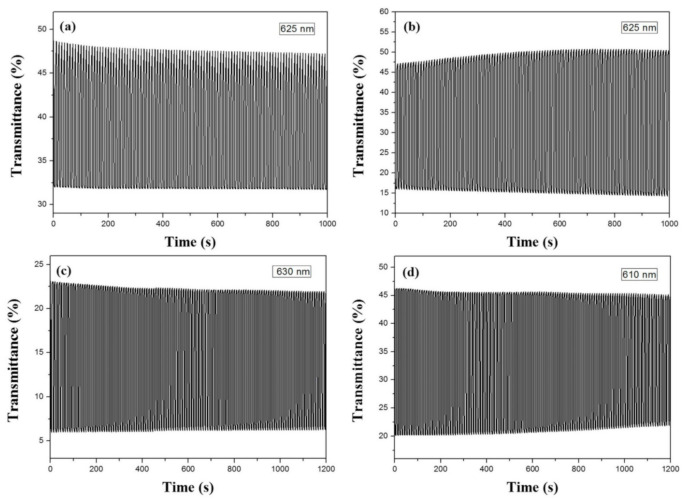
Optical contrast of (**a**) PBPBC/PEDOT, (**b**) P(BPBC-*co*-BT)/PEDOT, (**c**) P(BPBC-*co*-CDT)/PEDOT, and (**d**) P(BPBC-*co*-CDTK)/PEDOT ECDs with a residence time of 5 s.

Figure 11 displayed the coloring and bleaching kinetics of PBPBC/PEDOT, P(BPBC-*co*-BT)/PEDOT, P(BPBC-*co*-CDT)/PEDOT, and P(BPBC-*co*-CDTK)/PEDOT ECDs between 0.0 (the bleached state) and +1.8 V (the colored state) with a time interval of 5 s. The *τ*_c_ and *τ*_b_ of the four ECDs were summarized in Table 6, which were rapider than those of PBPBC, P(BPBC-*co*-BT), P(BPBC-*co*-CDT), and P(BPBC-*co*-CDTK) films in solutions. This can be ascribed to the distances between the two electrodes, which are short in ECDs [63]. In addition, the Δ*T*_max_ of PBPBC/PEDOT, P(BPBC-*co*-BT)/PEDOT, P(BPBC-*co*-CDT)/PEDOT, and P(BPBC-*co*-CDTK)/PEDOT ECDs were 17.0% at 625 nm, 36.2% at 625 nm, 17.3% at 630 nm, and 23.9% at 610 nm, respectively. The ECD using P(BPBC-*co*-BTP) as the anodic layer attained the highest Δ*T*_max_ among the four ECDs. Table 6 also summarized the *η* of the ECDs, which were 502.7, 418.3, 491.7, and 534.4 cm^2^ C^−1^ for PBPBC/PEDOT ECD at 625 nm, P(BPBC-*co*-BT)/PEDOT ECD at 625 nm, P(BPBC-*co*-CDT)/PEDOT ECD at 630 nm, and P(BPBC-*co*-CDTK)/PEDOT ECD at 610 nm, respectively. Based on the analysis of the above results, the ECD using a P(BPBC-*co*-CDTK) anodic layer attained the highest *η*.

Table 4 also summarizes the comparison of Δ*T* and *η* of P(BPBC-*co*-BT)/PEDOT ECD with the reported ECDs. P(BPBC-*co*-BT)/PEDOT ECD revealed a higher Δ*T* than that reported for P(dNCz-b)/PEDOT ECD at 700 nm [60], whereas the Δ*T* of P(BPBC-*co*-BT)/PEDOT ECD was comparable to those of poly(PS-Car)/PEDOT ECD at 640 nm [59] and P(DCP-*co*-CPDK)/PEDOT-PSS ECD at 635 nm [53]. Moreover, P(BPBC-*co*-BT)/PEDOT ECD showed a higher *η* than that reported for P(dNCz-b)/PEDOT ECD at 700 nm [60]. However, P(BPBC-*co*-BT)/PEDOT ECD showed a lower *η* than that reported for P(DCP-*co*-CPDK)/PEDOT-PSS ECD at 635 nm [53].

### 3.7. Optical Memory of ECDs

**Figure 12:**
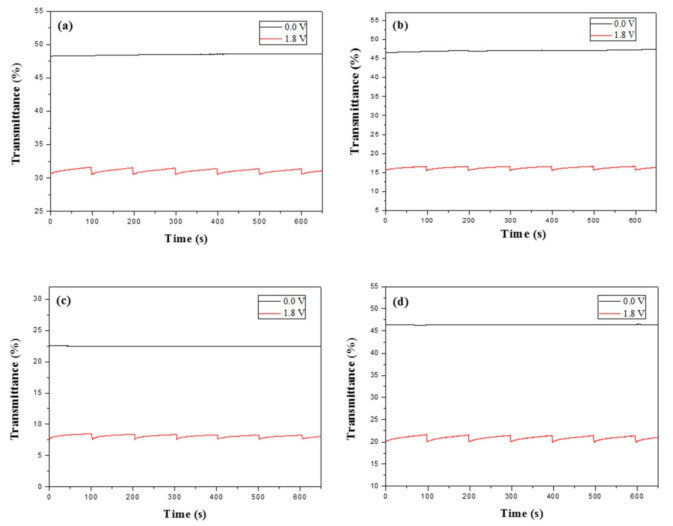
Open circuit stability of (**a**) PBPBC/PEDOT, (**b**) P(BPBC-*co*-BT)/PEDOT, (**c**) P(BPBC-*co*-CDT)/PEDOT, and (**d**) P(BPBC-*co*-CDTK)/PEDOT ECDs.

The transmittance-time diagrams of PBPBC/PEDOT, P(BPBC-*co*-BT)/PEDOT, P(BPBC-*co*-CDT)/PEDOT, and P(BPBC-*co*-CDTK)/PEDOT ECDs at bleached and colored states were monitored at 625, 625, 630, and 610 nm, respectively. The time for applying potentials in colored and bleached states was 1 s in each time interval of 100 s. As displayed in Figure 12, the transmittance variations of the four ECDs were ≤1% in the bleached (0.0 V) state and ≤4% in colored (+1.8 V) states, demonstrating that PBPBC/PEDOT, P(BPBC-*co*-BT)/PEDOT, P(BPBC-*co*-CDT)/PEDOT, and P(BPBC-*co*-CDTK)/PEDOT ECDs had sufficient optical circuit memory.

### 3.8. Redox Stability of ECDs

**Figure 13:**
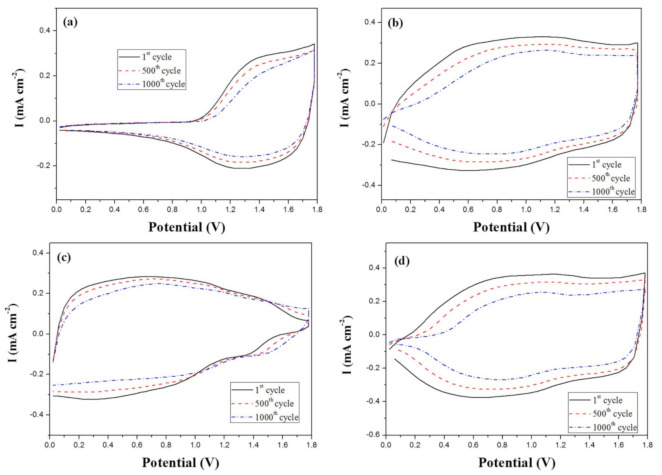
Cyclic voltammograms of (**a**) PBPBC/PEDOT, (**b**) P(BPBC-*co*-BT)/PEDOT, (**c**) P(BPBC-*co*-CDT)/PEDOT, and (**d**) P(BPBC-*co*-CDTK)/PEDOT devices at a scan rate of 500 mV s^−1^ between 1 and 1000 cycles.

The redox cycling stability of the four ECDs scanned between 0.0 V and +1.8 V was performed using a potentiodynamic electrochemical measurement [64,65]. As shown in Figure 13, the electroactivity of PBPBC/PEDOT, P(BPBC-*co*-BT)/PEDOT, P(BPBC-*co*-CDT)/PEDOT, and P(BPBC-*co*-CDTK)/PEDOT ECDs was 88.3%, 85.7%, 95.8%, and 83.6%, respectively, after sweeping between 0.0 and +1.8 V for the 500th cycle. 75.3%, 68.9%, 86.7%, and 61.2% of electroactivity was preserved after the 1000th cycle for PBPBC/PEDOT, P(BPBC-*co*-BT)/PEDOT, P(BPBC-*co*-CDT)/PEDOT, and P(BPBC-*co*-CDTK)/PEDOT ECDs, respectively. The results suggest that PBPBC and P(BPBC-*co*-CDT) are promising polymers for use as anodic layers in ECDs.

## 4. Conclusions

Four 1,3-bis(carbazol-9-yl)benzene-based polymers (PBPBC, P(BPBC-*co*-BT), P(BPBC-*co*-CDT), and P(BPBC-*co*-CDTK)) were electrodeposited using potentiodynamic methods. The four polymers showed quasi-reversible and multicolored properties. The P(BPBC-*co*-CDT) film displayed purplish-black, dark orchid, greyish-blue, and Prussian blue at 0.0, 0.4, 0.8, and 1.1 V, respectively. Electrochromic responding studies of polymeric electrodes showed that P(BPBC-*co*-BT) and P(BPBC-*co*-CDTK) displayed high transmittance changes of 44.4% at 1030 nm and 41.4% at 1070 nm, respectively. The *η* of PBPBC film was up to 286.9 cm^2^ C^–1^ at 430 nm. In addition, four ECDs consisting of four anodic electrochromic layers and a cathodic electrochromic layer were built. The P(BPBC-*co*-BT)/PEDOT and P(BPBC-*co*-CDTK)/PEDOT ECDs displayed transmittance variations of 36.2% at 625 nm and 23.9% at 610 nm, respectively. The PBPBC/PEDOT and P(BPBC-*co*-CDTK)/PEDOT ECDs realized *η* of 502.7 cm^2^ C^–1^ at 625 nm and 534.4 cm^2^ C^–1^ at 610 nm, respectively. The switching time of the four dual-type ECDs was less than 0.7 s. Given these results, PBPBC, P(BPBC-*co*-BT), P(BPBC-*co*-CDT), and P(BPBC-*co*-CDTK) are promising candidates as electrodes for ECDs.

## Figures and Tables

**Table 1:** Feed species of anodic polymers.

Electrodes	Anodic Polymers	Feed Species	Feed Molar Ratio
(a)	PBPBC	2 mM BPBC	Neat BPBC
(b)	P(BPBC-*co*-BT)	2 mM BPBC + 2 mM BT	BPBC:BT = 1:1
(c)	P(BPBC-*co*-CDT)	2 mM BPBC + 2 mM CDT	BPBC:CDT = 1:1
(d)	P(BPBC-*co*-CDTK)	2 mM BPBC + 2 mM CDTK	BPBC:CDTK = 1:1

**Table 2:** Colorimetric values (L*, a*, and b*), CIE chromaticity values (*x*, *y*), and diagrams of (**a**) PBPBC, (**b**) P(BPBC-*co*-BT), (**c**) P(BPBC-*co*-CDT), and (**d**) P(BPBC-*co*-CDTK) at several potentials.

Films	*E* (V)	Graphs	L*	a*	b*	*x*	*y*	Diagrams
**(a)**	0.0	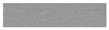	89.69	0.54	7.65	0.3276	0.3435	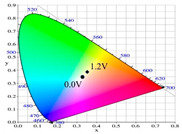
	0.4	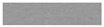	89.95	0.65	7.67	0.3278	0.3434	
	0.8	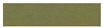	89.99	0.53	7.77	0.3278	0.3437	
	1.2	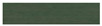	85.23	−5.22	24.64	0.3517	0.3837	
**(b)**	0.0	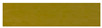	80.69	2.86	52.78	0.4186	0.4315	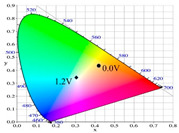
	0.4	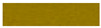	80.57	3.10	52.46	0.4186	0.4307	
	0.8	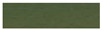	75.87	−6.82	34.12	0.3716	0.4118	
	1.2	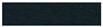	65.37	−7.54	1.76	0.303	0.3403	
**(c)**	0.0	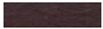	55.12	−11.16	0.4	0.2904	0.3415	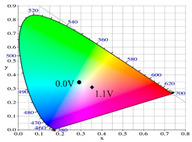
	0.4	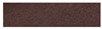	51.72	−4.78	−4.28	0.2901	0.3208	
	0.8	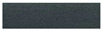	51.92	5.07	6.13	0.3418	0.3416	
	1.1	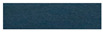	40.76	16.2	−0.97	0.3519	0.3055	
**(d)**	0.0	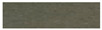	70.51	−6.24	18.29	0.3424	0.379	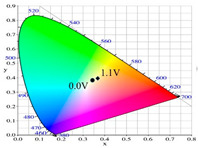
	0.4	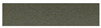	70.68	−5.98	18.03	0.3423	0.378	
	0.8	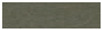	70.13	−2.74	21.85	0.3568	0.3839	
	1.1	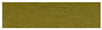	72.39	−3.64	27.8	0.3667	0.3971	

**Table 3:** Optical and electrochromic switching properties investigated at selected wavelengths for the electrodes.

Electrodes	λ (nm)	*T*_ox_	*T*_red_	Δ*T*	ΔOD	*Q_d_* (mC cm^−2^)	*η* (cm^2^ C^−1^)	*τ*_c_ (s)	*τ*_b_ (s)
PBPBC	1040	47.9	77.5	29.6	0.209	1.49	140.3	1.3	2.2
	430	50.6	58.9	8.3	0.066	0.23	286.9	0.4	1.5
P(BPBC-*co*-BT)	1030	15.5	59.9	44.4	0.586	4.54	130.0	1.2	2.5
	680	15.3	53.2	37.9	0.541	2.48	218.1	1.0	2.5
P(BPBC-*co*-CDT)	1050	4.8	27.1	22.3	0.752	6.18	121.7	2.1	2.4
	780	9.3	22.1	12.8	0.376	4.92	76.4	2.5	1.5
P(BPBC-*co*-CDTK)	1070	12.8	54.2	41.4	0.627	2.21	283.7	2.4	2.5
	460	19.2	22.9	3.7	0.077	0.27	285.2	0.7	2.3

**Table 4:** Transmittance variations and *η* of polymers and ECDs.

Polymer Films or ECD Configurations	*λ* (nm)	Δ*T* (%)	*η* (cm^2^ C^−1^)	References
P(DiCP-*co*-CPTK2)	890	35.9	111.5	[53]
PITD-2	675	18	171.5	[54]
DPPA-2SNS	900	58	224	[56]
P2	779	36	123	[55]
PI-6A	573	55	191	[57]
P(BPBC-*co*-BT)	680	37.9	218	This work
Poly(PS-Car)/PEDOT	640	38	-	[59]
P(dNCz-b)/PEDOT	700	29	234	[60]
P(DCP-*co*-CPDK)/PEDOT-PSS	635	38.2	634	[53]
P(BPBC-*co*-BT)/PEDOT	625	36.2	418.3	This work

**Table 5:** Electrochromic photographs, colorimetric values (L*, a*, and b*), and CIE chromaticity values (*x*, *y*) of PBPBC/PEDOT, P(BPBC-*co*-BTP)/PEDOT, P(BPBC-*co*-CDT)/PEDOT, and P(BPBC-*co*-CDTK)/PEDOT ECDs at several potentials.

Devices	*E* (V)	Graphs	L*	a*	b*	*x*	*y*	Diagrams
PBPBC/PEDOT	0.0	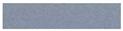	79.33	−6.79	1.94	0.3060	0.3384	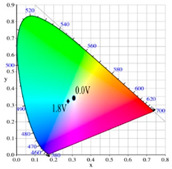
	0.8	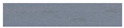	77.84	−7.08	3.31	0.3082	0.3419	
	1.2	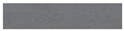	72.84	−5.96	4.36	0.3121	0.3442	
	1.6	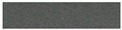	63.78	−8.8	3.07	0.3035	0.3452	
	2.0	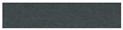	55.48	−9.38	−6.66	0.2758	0.3183	
P(BPBC-*co*-BT)/PEDOT	0.0	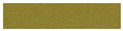	72.77	−8.24	28.15	0.3587	0.4029	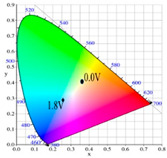
	0.8	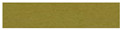	70.50	−10.32	21.94	0.3427	0.3923	
	1.2	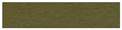	63.34	−9.55	6.42	0.3101	0.3552	
	1.6	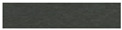	53.95	−6.52	−9.9	0.2722	0.3058	
	1.8	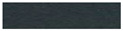	49.28	−4.87	−16.34	0.255	0.2827	
P(BPBC-*co*-CDT)/PEDOT	−0.6	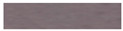	52.80	2.58	2.58	0.3257	0.3339	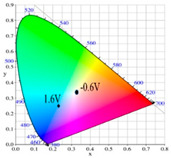
	0.6	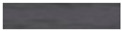	52.20	−5.74	−3.51	0.2904	0.3241	
	1.0	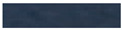	48.44	−5.15	−12.69	0.2639	0.2937	
	1.4	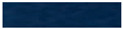	42.77	−2.24	−21.43	0.2396	0.259	
	1.6	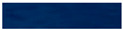	40.27	−1.12	−24.76	0.2291	0.2444	
P(BPBC-*co*-CDTK)/PEDOT	0.0	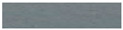	73.76	−7.83	9.04	0.3189	0.3568	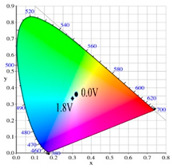
	0.8	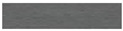	71.1	−5.13	14.31	0.3355	0.3679	
	1.2	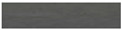	66.53	−3.9	14.07	0.3385	0.3681	
	1.6	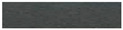	59.79	−4.51	5.17	0.3169	0.3477	
	1.8	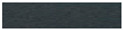	54.81	−4.87	−1.3	0.2989	0.3301	

**Table 6:** Optical and electrochromic switching properties investigated at selected wavelengths for the devices.

ECDs	*λ* (nm)	*T*_ox_	*T*_red_	Δ*T*	ΔOD	*Q_d_* (mC cm^−2^)	*η* (cm^2^∙C^−1^)	*τ*_c/_s	*τ*_b/_s
PBPBC/PEDOT	625	31.9	48.9	17.0	0.186	0.37	502.7	0.7	0.6
P(BPBC-*co*-BT)/PEDOT	625	14.3	50.5	36.2	0.548	1.31	418.3	0.5	0.4
P(BPBC-*co*-CDT)/PEDOT	630	5.9	23.2	17.3	0.595	1.21	491.7	0.4	0.6
P(BPBC-*co*-CDTK)/PEDOT	610	21.4	45.3	23.9	0.326	0.61	534.4	0.2	0.4

## Data Availability

The data presented in this study are available on request from the corresponding author.

## Supplementary Materials

The following are available online at https://www.mdpi.com/article/10.3390/polym13071136/s1, Figure S1: FT-IR spectra of (a) PBPBC, (b) P(BPBC-*co*-BT), (c) P(BPBC-*co*-CDT), and (d) P(BPBC-*co*-CDTK), Figure S2: The NMR tubes of PBPBC, P(BPBC-*co*-BT), P(BPBC-*co*-CDT), and P(BPBC-*co*-CDTK) in CD_2_Cl_2_ and CF_3_COOD solvents. More than 95% polymer samples are insoluble in CD_2_Cl_2_ and CF_3_COOD, Figure S3: The ^1^H NMR spectra of (a) BPBC, (b) BT, (c) CDT, and (d) CDTK in deuterated solvent, Figure S4: The ^1^H NMR spectra of (a) PBPBC, (b) P(BPBC-*co*-BT), (c) P(BPBC-*co*-CDT), and (d) P(BPBC-*co*-CDTK) in CD_2_Cl_2_. Less than 5% polymer samples are soluble in deuterated solvent, the ^1^H NMR results are not clearly indicate all structures of polymers. More than 95% insoluble polymer samples are not presented in ^1^H NMR spectra, Figure S5: Transmittance-time profiles of PBPBC under (a) dark environment and (b) AM 1.5 irradiation (100 mW cm^−2^) with a residence time of 5 s. The measurements were carried out after under dark (or light) condition for 30 h, Figure S6: Charge-time plots of (a) PBPBC/PEDOT, (b) P(BPBC-*co*-BT)/PEDOT, (c) P(BPBC-*co*-CDT)/PEDOT, and (d) P(BPBC-*co*-CDTK)/PEDOT ECDs with a residence time of 5 s.
