# Supplementary material for: Electrodeposited Copolymers Based on 9,9′-(5-Bromo-1,3-phenylene)biscarbazole and Dithiophene Derivatives for High-Performance Electrochromic Devices
Source: Polymers (Basel). 2021 Apr 2;13(7):1136. doi: 10.3390/polym13071136 (PMC8038177; doi:10.3390/polym13071136)
Supplement: Supplementary file 1 [file polymers-13-01136-s001.pdf]

Supplementary Information

Electrodeposited Copolymers Based on 9,9′-(5-Bromo-1,3-Phenylene)Biscarbazole and Dithiophene Derivatives for High-Performance Electrochromic Devices

Chung-Wen Kuo [1], Jui-Cheng Chang [2,3], Jeng-Kuei Chang [4], Sheng-Wei Huang [1], Pei-Ying Lee [2] and Tzi-Yi Wu [2,*]

1. Department of Chemical and Materials Engineering, National Kaohsiung University of Science and Technology, Kaohsiung 80778, Taiwan; welly@nkust.edu.tw (C.-W.K.); bill4794@gmail.com (S.-W.H.)
2. Department of Chemical Engineering and Materials Engineering, National Yunlin University of Science and Technology, Yunlin 64002, Taiwan; d700215@gmail.com (J.-C.C.); leepeiying1018@gmail.com (P.-Y.L.)
3. Bachelor Program in Interdisciplinary Studies, National Yunlin University of Science and Technology, Yunlin 64002, Taiwan
4. Department of Materials Science and Engineering, National Yang Ming Chiao Tung University, No. 1001 University Road, Hsinchu 30010, Taiwan; jkchang@nctu.edu.tw

* Correspondence: wuty@gemail.yuntech.edu.tw; Tel.: +886-5-534-2601 (ext. 4626)

Figure S1 showed the infrared spectra of PBPBC, P(BPBC-*co*-BT), P(BPBC-*co*-CDT), and P(BPBC-*co*-CDTK) films. As displayed in Figure S1(a), the infrared peak at 1065 cm^{-1} implied the doping of PBPBC with the electrolyte (ClO_4^-). The infrared peak at ca. 1593 cm^{-1} stands for the C=C stretching vibration of phenyl group. The peak at ca. 1451 cm^{-1} can be attributed to the C–N stretching of the carbazole group.

There was no noticeable -C-S-C- stretching peak of PBPBC at around 790-803 cm^{-1}. Figure S1b–d displayed the infrared peaks of PBPBC and -C-S-C- stretching vibration of P(BPBC-*co*-BT), P(BPBC-*co*-CDT), and P(BPBC-*co*-CDTK), the presence of new peaks at 790, 803, and 797 cm^{-1} could be attributed to the existence of BT, CDT and CDTK in P(BPBC-*co*-BT), P(BPBC-*co*-CDT), and P(BPBC-*co*-CDTK) films, respectively. The peak at ca. 1708 cm^{-1} can be ascribed to the C=O stretching of P(BPBC-*co*-CDTK).

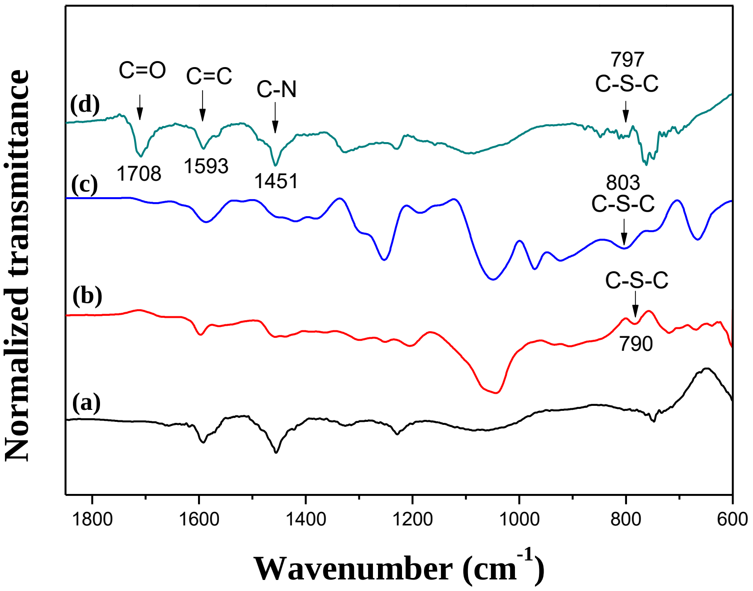

Figure S1. FT-IR spectra of (a) PBPBC, (b) P(BPBC-*co*-BT), (c) P(BPBC-*co*-CDT), and (d) P(BPBC-*co*-CDTK).

CD_2Cl_2 and CF_3COOD are deuterated solvents with strong dissolving ability. CD_2Cl_2 and CF_3COOD solvents are used to dissolve as-prepared polymer films. However, only less than 5% as-prepared polymer films are soluble in CD_2Cl_2 and CF_3COOD (Figure S2).

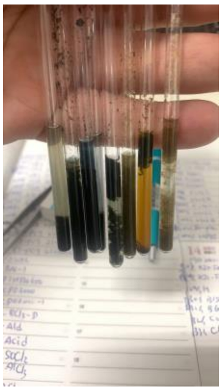

Figure S2. The NMR tubes of PBPBC, P(BPBC-*co*-BT), P(BPBC-*co*-CDT), and P(BPBC-*co*-CDTK) in CD_2Cl_2 and CF_3COOD solvents. More than 95% polymer samples are insoluble in CD_2Cl_2 and CF_3COOD.

(a)

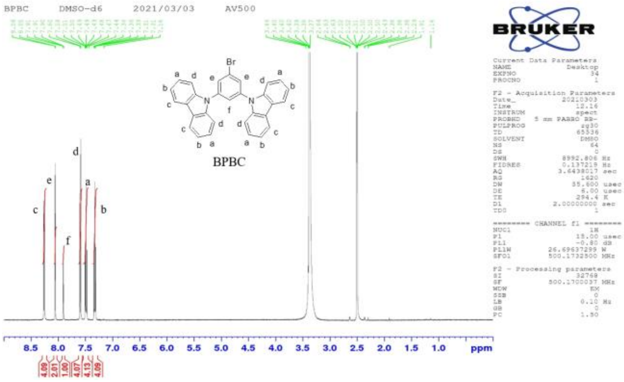

(b)

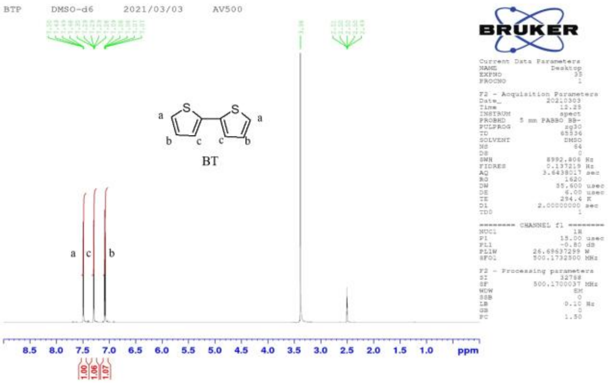

(c)

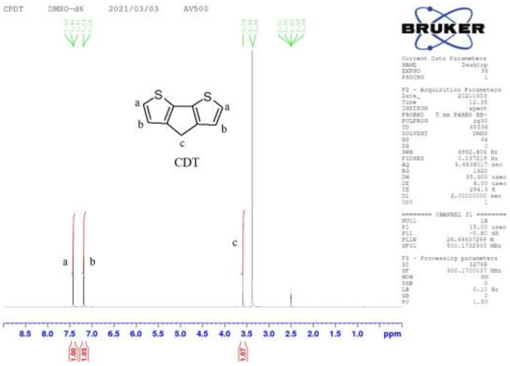

(d)

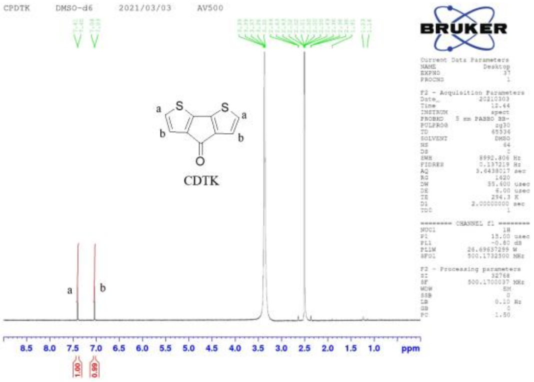

Figure S3. The ^1H NMR spectra of (a) BPBC, (b) BT, (c) CDT, and (d) CDTK in deuterated solvent.

The ^1H NMR spectrum does not display any significant signal for less than 5% soluble polymers in CF_3COOD. Nevertheless, less than 5% soluble polymers in CD_2Cl_2 reveal some weak ^1H NMR signals. Figure S3 shows the ^1H NMR spectra of monomers (BPBC, BT, CDT, and CDTK) and Figure S4 shows the ^1H NMR spectra of less than 5% soluble polymers (PBPBC, P(BPBC-*co*-BT), P(BPBC-*co*-CDT), and P(BPBC-*co*-CDTK)) in

deuterated solvents. The ^{1}H NMR peaks of partial soluble polymers are broader than those of monomers in deuterated solvents, indicating the formation of polymers after electrochemical polymerization. However, less than 5% polymer samples are soluble in CD_2Cl_2, the ^{1}H NMR results do not clearly indicate all structures of polymers. The ^{1}H NMR spectra only display the signals of soluble bicarbazole-based homomers. Bithiophene-based homopolymers (PBT, PCDT, and PCDTK) and three copolymers (P(BPBC-*co*-BT), P(BPBC-*co*-CDT), and P(BPBC-*co*-CDTK)) are almost insoluble in CD_2Cl_2. More than 95% insoluble polymer samples are not presented in ^{1}H NMR spectra.

(a)

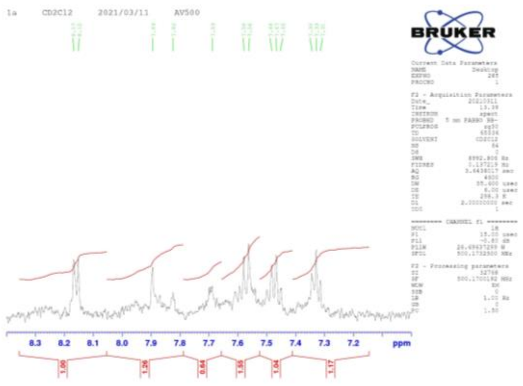

(b)

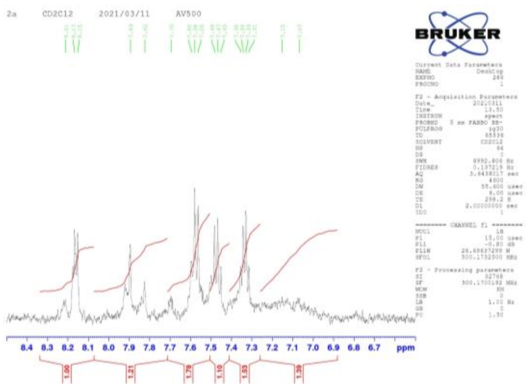

(c)

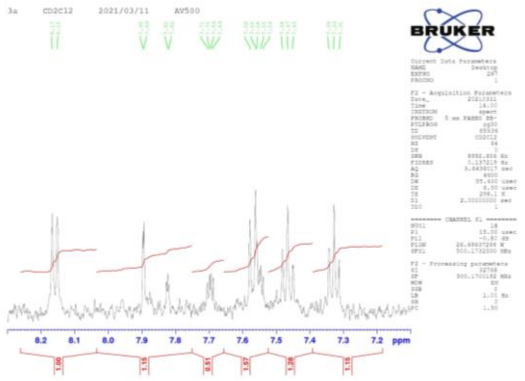

(d)

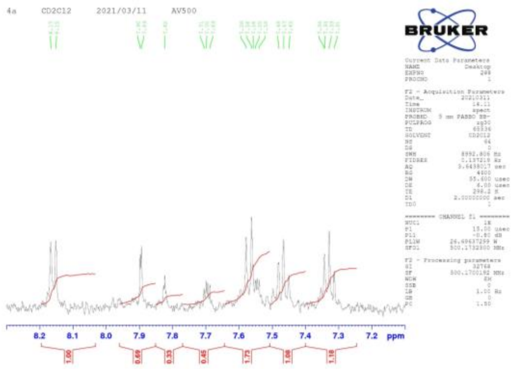

Figure S4. The ^{1}H NMR spectra of (a) PBPBC, (b) P(BPBC-*co*-BT), (c) P(BPBC-*co*-CDT), and (d) P(BPBC-*co*-CDTK) in CD_2Cl_2. Less than 5% polymer samples are soluble in deuterated solvent, the ^{1}H NMR results are not clearly indicate all structures of polymers. More than 95% insoluble polymer samples are not presented in ^{1}H NMR spectra.

Figure S5 showed the electrochromic switching plot of PBPBC after under dark (or light) environment for 30 hr, the ΔT of PBPBC under light conditions was 98.7% of that under dark conditions. It implied that PBPBC revealed sufficient ΔT stability when exposed to light.

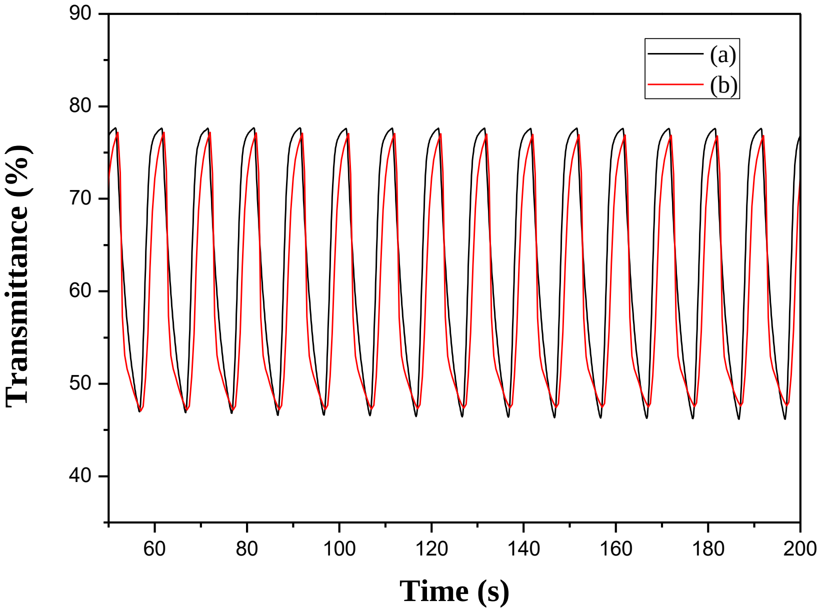

Figure S5. Transmittance-time profiles of PBPBC under (a) dark environment and (b) AM 1.5 irradiation (100 mW cm^{-2}) with a residence time of 5 s. The measurements were carried out after under dark (or light) condition for 30 h.

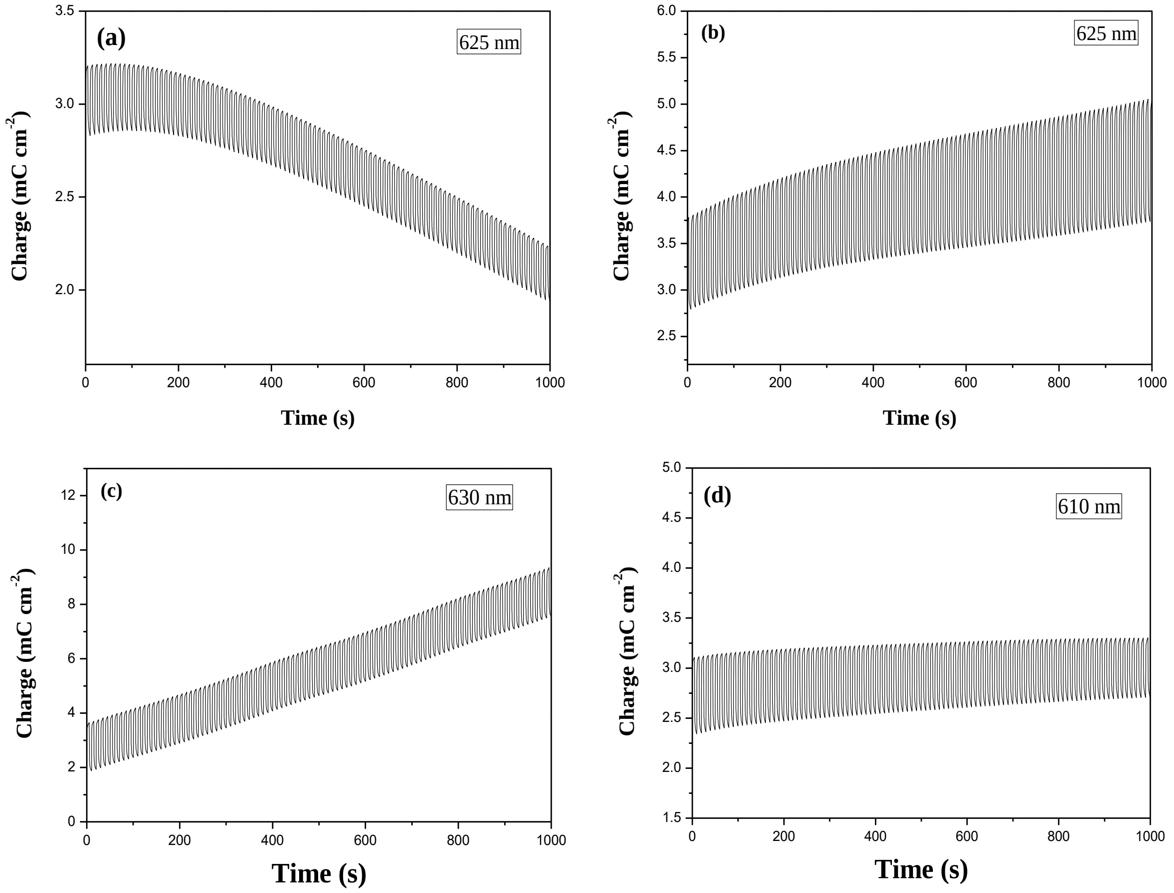

Figure S6. Charge-time plots of (a) PBPBC/PEDOT, (b) P(BPBC-*co*-BT)/PEDOT, (c) P(BPBC-*co*-CDT)/PEDOT, and (d) P(BPBC-*co*-CDTK)/PEDOT ECDs with a residence time of 5 s.
